# Attempting to Identify Bacterial Allies in Immunotherapy of NSCLC Patients

**DOI:** 10.3390/cancers14246250

**Published:** 2022-12-19

**Authors:** Anna Grenda, Ewelina Iwan, Paweł Krawczyk, Małgorzata Frąk, Izabela Chmielewska, Arkadiusz Bomba, Aleksandra Giza, Anna Rolska-Kopińska, Michał Szczyrek, Robert Kieszko, Tomasz Kucharczyk, Bożena Jarosz, Dariusz Wasyl, Janusz Milanowski

**Affiliations:** 1 Department of Pneumonology, Oncology and Allergology, Medical University of Lublin, Jaczewskiego 8, 20-950 Lublin, Poland; 2 Department of Omics Analyses, National Veterinary Research Institute, Partyzantow 57, 24-100 Pulawy, Poland; 3 Department of Neurosurgery and Pediatric Neurosurgery, Medical University of Lublin, Jaczewskiego 8, 20-950 Lublin, Poland

**Keywords:** non-small cell lung cancer, microbiome, *Clostridia*, immunotherapy

## Abstract

**Simple Summary:**

One of the newest therapies for the treatment of non-small cell lung cancer is immunotherapy targeting the immune checkpoints PD-1 and PD-L1. Factors are now being sought to predict the efficacy of this treatment, as PD-L1 expression on tumor cells is not a perfect indicator. In this aspect, the lineup of the gut microbiome of patients treated with immunotherapy draws attention. Our study was designed to investigate how the presence of bacteria from certain groups is associated with the effectiveness of immunotherapy or chemoimmunotherapy. The results of studies such as ours presented here may help optimize the qualification of patients with non-small cell lung cancer for treatment with immune checkpoint inhibitors.

**Abstract:**

Introduction: Factors other than PD-L1 (Programmed Death Ligand 1) are being sought as predictors for cancer immuno- or chemoimmunotherapy in ongoing studies and long-term observations. Despite high PD-L1 expression on tumor cells, some patients do not benefit from immunotherapy, while others, without the expression of this molecule, respond to immunotherapy. Attention has been paid to the composition of the gut microbiome as a potential predictive factor for immunotherapy effectiveness. Materials and Methods: Our study enrolled 47 Caucasian patients with stage IIIB or IV non-small cell lung cancer (NSCLC). They were eligible for treatment with first- or second-line immunotherapy or chemoimmunotherapy. We collected stool samples before the administration of immunotherapy. We performed next-generation sequencing (NGS) on DNA isolated from the stool sample and analyzed bacterial V3 and V4 of the *16S rRNA* gene. Results: We found that bacteria from the families *Barnesiellaceae*, *Ruminococcaceae*, *Tannerellaceae*, and *Clostridiaceae* could modulate immunotherapy effectiveness. A high abundance of *Bacteroidaaceae*, *Barnesiellaceae,* and *Tannerellaceae* could extend progression-free survival (PFS). Moreover, the risk of death was significantly higher in patients with a high content of *Ruminococcaceae* family (HR = 6.3, 95% CI: 2.6 to 15.3, *p* < 0.0001) and in patients with a low abundance of *Clostridia UCG-014* (HR = 3.8, 95% CI: 1.5 to 9.8, *p* = 0.005) regardless of the immunotherapy line. Conclusions: The *Clostridia* class in gut microbiota could affect the effectiveness of immunotherapy, as well as the length of survival of NSCLC patients who received this method of treatment.

## 1. Introduction

Currently, the blockade of immune checkpoints by immune checkpoint inhibitors (ICIs) is used in the standard immunotherapy of patients with advanced non-small cell lung cancer (NSCLC). The targets for immunotherapy in NSCLC patients are PD-L1 (Programmed Cell Death-Ligand 1), PD-1 (Programmed Cell Death 1), and CTLA-4 (Cytotoxic T Lymphocyte Antigen 4) [1,2]. ICIs are used in monotherapy or in combination with chemotherapy, and in patients with advanced NSCLC, they significantly prolong survival. The toxicity of antibodies anti-PD1, anti-PD-L1, or anti-CTLA-4 is at an acceptable level [3,4,5,6]. Pembrolizumab in first-line monotherapy compared to platinum compound-based chemotherapy prolongs overall survival (OS) and progression-free survival (PFS) in patients with locally advanced or advanced NSCLC with PD-L1 expression on at least 1% of tumor cells [5]. However, in UE countries, pembrolizumab monotherapy is used in patients with PD-L1 expression on ≥50% of tumor cells according to the results of the KEYNOTE 024 clinical trial. Moreover, cemiplimab can also be used in this indication [7]. Atezolizumab monotherapy significantly prolongs overall survival compared to platinum-based chemotherapy among patients with NSCLC with high PD-L1 expression (≥50% of tumor cells or ≥10% of tumor-infiltrating immune cells) [4]. For previously untreated metastatic non-squamous cell carcinoma, the use of chemoimmunotherapy with pembrolizumab plus pemetrexed and platinum-based compounds prolongs OS and PFS compared to chemotherapy alone [8]. Pembrolizumab with chemotherapy combined with paclitaxel or nab-paclitaxel could be used in squamous cell carcinoma patients. Moreover, combined treatment with nivolumab, ipilimumab, and limited chemotherapy (two cycles) finds application in the treatment of advanced NSCLC [9]. Chemoimmunotharpies result in improved PFS and OS regardless of PD-L1 expression or the presence of liver or brain metastases, although the lack of PD-L1 expression or the occurrence of metastases reduces the effectiveness of therapy [3]. In immunotherapy-naive patients who have received first-line chemotherapy, nivolumab or atezolizumab second-line monotherapy is used regardless of PD-L1 expression on tumor cells. In such patients, pembrolizumab is applied if ≥1% of tumor cells express PD-L1 [10]. In ongoing studies and long-term observations on the efficacy of immuno- or chemoimmunotherapy, factors other than PD-L1 expression are being sought as predictive factors. This is because some patients, despite high PD-L1 expression, do not benefit from ICIs treatment, while patients without expression of this molecule on tumor cells show long-term responses to immunotherapy [11,12].

The attention of researchers has been directed to the composition of the gut microbiome as a potential predictor of immunotherapy effectiveness [13]. Diet, antibiotics, and age can modulate gut microbiota. The relationship between the course of several diseases and microbiota composition has been reported multiple times [14]. Resident bacteria in the intestine can stimulate the immune system by modulating cytotoxic or helper T lymphocytes [15,16]. Bacteria produce several substances that can affect the effectiveness of immunotherapy or render it ineffective. Analysis of the microbiome composition across a broad spectrum is possible thanks to the development of next-generation sequencing (NGS) techniques. NGS allows the direct identification of bacterial composition in environmental samples at the Phylum level and individual species. Literature data indicate a whole range of bacteria that favor the effectiveness of immunotherapy and may be involved in its failure [17,18,19,20,21,22]. Bacteria such as *Akkermansia mucinifila*, *Enterococcus hirae,* and *Barnesiella intestinihominis*, or bacteria whose taxonomic status has been distinguished relatively recently through the use of NGS, are mentioned. Depending on the type of cancer, the same bacteria can be identified as associated with the efficacy or lack of effectiveness of immunotherapy [22,23].

In our study, we analyzed the intestinal microbiome composition of patients with advanced NSCLC and assessed how this composition affects the efficacy of ICIs treatment in monotherapy or combination with chemotherapy.

## 2. Materials and Methods

### 2.1. Studied Group

From December 2018 to July 2020, we collected fecal samples from patients treated with immunotherapy at the Department of Pneumonology, Oncology, and Allergology at the Medical University of Lublin. There were 17 (36%) women and 30 (64%) men. The median age was 66 years (min–max: 49–79 years). The study group consisted of 47 Caucasian patients with stage IIIB or IV NSCLC. Twenty-eight (60%) patients were diagnosed with adenocarcinoma (AC), while 19 patients (40%) were diagnosed with squamous cell carcinoma (SqC). Forty (85%) patients were current or former smokers, while 7 patients (15%) had never smoked cigarettes. Ten patients (21%) received antibiotic therapy for up to 4 (1–4) weeks before treatment. The percentage of tumor cells PD-L1 expression was determined in all patients by routine diagnostic immunohistochemistry (with SP 263 monoclonal antibody, Ventana, Roche Diagnostics). Thirty-one patients (66%) had the expression of PD-L1 on <50% of tumor cells, while 16 patients (34%) had PD-L1 expression on ≥50% of tumor cells. In patients with AC, the presence of mutations in the *EGFR* (Epidermal Growth Factor Receptor) gene and rearrangement of the *ALK* (Anaplastic Lymphoma Kinase) and ROS1 (ROS proto-oncogene 1) genes were excluded.

Patients were qualified for treatment with first-line (pembrolizumab, n = 12, 25%) or second-line (nivolumab or atezolizumab, n = 35, 75%) immunotherapy. Patients treated with first-line chemotherapy received the following regimens: Platinum compounds + vinorelbine (n = 17, 48%), platinum compounds + pemetrexed (n = 15, 43%), platinum compound + taxane, (n = 1, 3%), pemetrexed in monotherapy (n = 1, 3%), and platinum compound in monotherapy (n = 1, 3%). Ten patients received palliative radiotherapy before chemotherapy.

Fourteen patients (30%) experienced various organ toxicities associated with immunotherapy treatment: Skin toxicity (n = 1, 2%), hyperthyroidism (n = 1, 2%), pneumonia (n = 4, 8.5%), liver toxicity (n = 6, 13%), joint toxicity (n = 2, 4%), and intestinal and pancreatic toxicity (n = 1, 2%). All toxicities were grade 1–2 according to CTCAE (Common Terminology Criteria for Adverse Events) and they did not require treatment discontinuation.

Informed consent was obtained from patients for the study. The study was approved by the local Bioethics Committee at the Medical University of Lublin (approval number—KE-0254/95/2018).

### 2.2. Sample Collection and Sequencing

Fecal samples were self-collected by patients residing in the clinic during qualification for immunotherapy. Stool samples collected before treatment administration were frozen immediately after collection until DNA isolation. The isolation procedure was previously described [24]. The obtained isolates were used to create libraries and then for sequencing as previously described [24]. Libraries have been prepared according to the 16S metagenomics protocol (Illumina). Their quality was checked by gel electrophoresis with using Fragment Analyzer (Agilent, Santa Clara, CA, USA) and dsDNA 935 Reagent Kit (Agilent, California, CA, USA). Normalization of the libraries was carried out using a fluorimeter Qubit 3.0 (Thermo Fisher Scientific, Waltham, MA, USA) and High Sensitivity Assay (Thermo Fisher Scientific, Waltham, MA, USA). Normalized libraries were used for sequencing on MiSeq (Illumina, San Diego, CA, USA) by pair-end sequencing (2 × 300 bp with V3 kit, Illumina). As a result of sequencing, FastQ files were generated and quality was checked with FastQC. The analyzed regions were V3 and V4 of the 16S rRNA gene, where the bacterial genome is the greatest nucleotide diversity, characteristic of individual bacterial groups. This property allows them to be distinguished. The analyzed regions were assets via Qiime 2.0 with Silva 138 database.

### 2.3. Statistical Analysis

The U-Mann-Whitney test was used to analyze independent groups for comparisons of differences in percentages of bacteria abundance concerning grouping variables. Kaplan–Meier analysis was used to compare survival times between groups with high (above the median) and low (below the median) percentages of individual bacteria groups. The Cox regression model with a stepwise selection procedure was used to establish a predictive model for patients treated with immunotherapy regardless of the line of treatment. We performed the analysis using Statistica 13 (TIBCO Software, Palo Alto, CA, USA) and MedCalc (MedCalc Software Ltd., Ostend, Belgium) software. The *p*-value was considered significant if it was less than 0.05.

## 3. Results

### 3.1. Antibiotic Therapy before Immunotherapy

**Figure 1:**
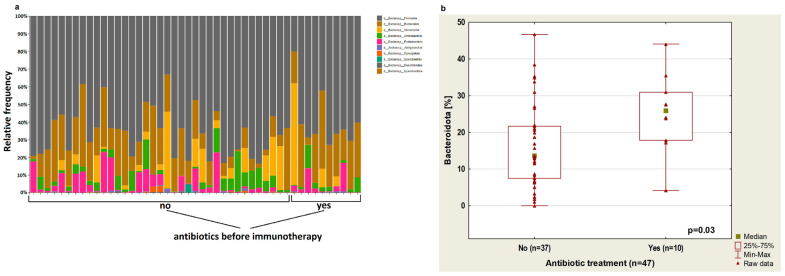
(**a**) Plot showing the relative frequency of bacterial phylum based on 16S rRNA of 47 stool samples from NSCLC patients treated or not treated with antibiotics; (**b**) boxplot showing *Bacteroidota* content depending on antibiotic therapy up to 4 weeks before immunotherapy.

Figure 1a shows the relative frequency of bacterial phylum based on 16S rRNA of 47 stool samples from NSCLC patients treated or not treated with antibiotics.

**Figure 2:**
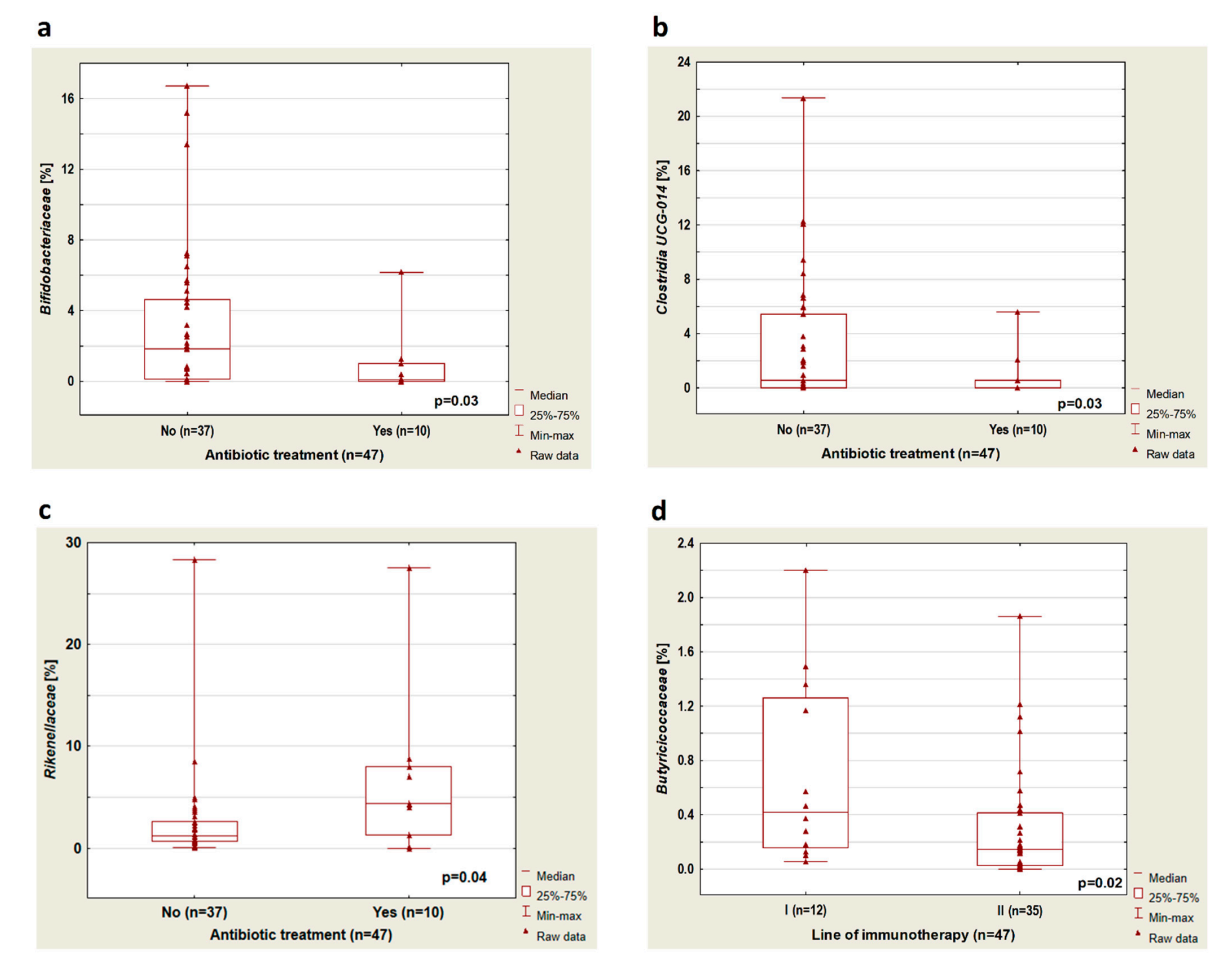
Boxplots showing the content of (**a**) *Bifidobacteriaceae*, (**b**) *Clostridia UCG-014*, (**c**) *Rikenellaceae* depending on the application of antibiotic treatment, and (**d**) *Butiriciococcaceae* depending on the line of immunotherapy.

The analysis showed that treatment with antibiotics up to 4 weeks before the immunotherapy cause an increase in Bacteroidota abundance (*p* = 0.03, Figure 1b). In patients treated with antibiotics before immunotherapy, there was a reduced abundance of bacteria from the *Bifidobacteriaceae* group (*p* = 0.03) and *Clostridia UCG-14* (*p* = 0.03). Furthermore, in this group of patients, an increased percentage of *Rikenellaceae* (*p* = 0.04) was observed (Figure 2a–c, respectively) regardless of immunotherapy lines.

### 3.2. Line of Immunotherapy

**Figure 3:**
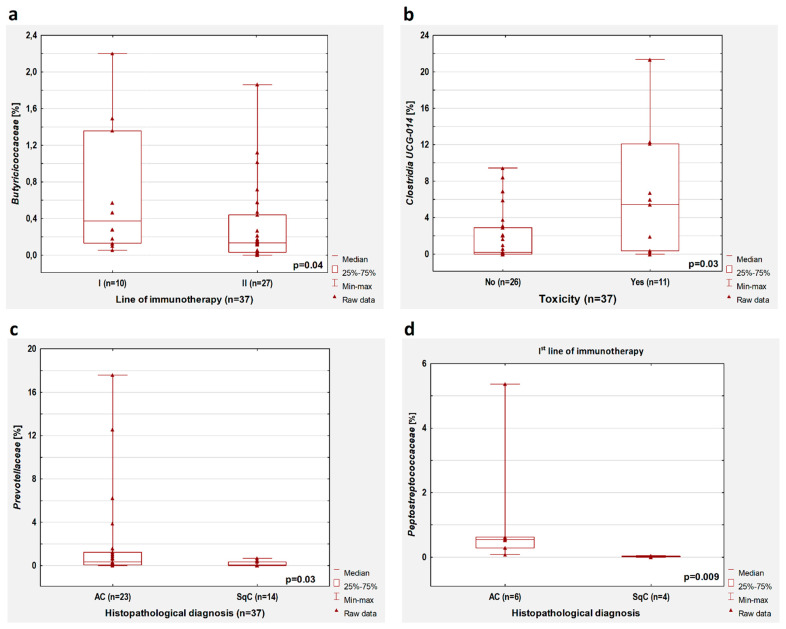
Boxplots showing the content of (**a**) *Butiriciococcaceae* depending on the line of immunotherapy; (**b**) *Clostridia UCG-014* depending on immunotherapy toxicity; (**c**) *Prevotellaceae* depending on the histopathological diagnosis; (**d**) *Peptostreptococcaceae* depending on the histopathological diagnosis in patients who received first-line immunotherapy. All results concern the patients untreated with antibiotics before immunotherapy.

A higher percentage of bacteria from the group of *Butyriciococcaceae* was observed in patients treated with immunotherapy in the first-line therapy compared to patients treated with ICIs in the second-line therapy (*p* = 0.02, Figure 2d). Furthermore, we performed a microbiome composition analysis in a group of previously untreated antibiotics (n = 37). In this group, we found that in patients treated with immunotherapy in the second-line therapy compared to patients who received first-line immunotherapy, there was a lower percentage of *Butyricicoccaceae* (*p* = 0.04, Figure 3a).

### 3.3. Toxicity of Immunotherapy

In the group without antibiotic treatment, we observed that the occurrence of immunotherapy toxicity was associated with a higher percentage of *Clostridia UCG-014* (*p* = 0.03, Figure 3b). However, this significant dependence was observed only in patients treated with first-line immunotherapy (*p* = 0.03) but not in patients who received second-line immunotherapy (*p* > 0.05). We did not confirm these observations in the whole group of patients (with and without antibiotic therapy).

### 3.4. Histopathologic Diagnosis

In patients without antibiotic therapy, lower intestinal content of *Prevotellaceae* (*p* = 0.03, Figure 3c) was found in patients with squamous cell carcinoma compared to patients with adenocarcinoma. This observation concerned patients treated with first-line immunotherapy (*p* = 0.009) but not patients who received second-line immunotherapy (*p* > 0.05). Moreover, adenocarcinoma patients treated with first-line immunotherapy had a higher content of *Peptostreptococcaceae* compared to squamous cell carcinoma patients (*p* = 0.009, Figure 3d). In patients treated with first-line immunotherapy regardless of previous antibiotic therapy history, the abundance of *Peptostreptococcaceae* was also higher in adenocarcinoma patients than in SqC patients (*p* = 0.005).

### 3.5. Response to Treatment

**Figure 4:**
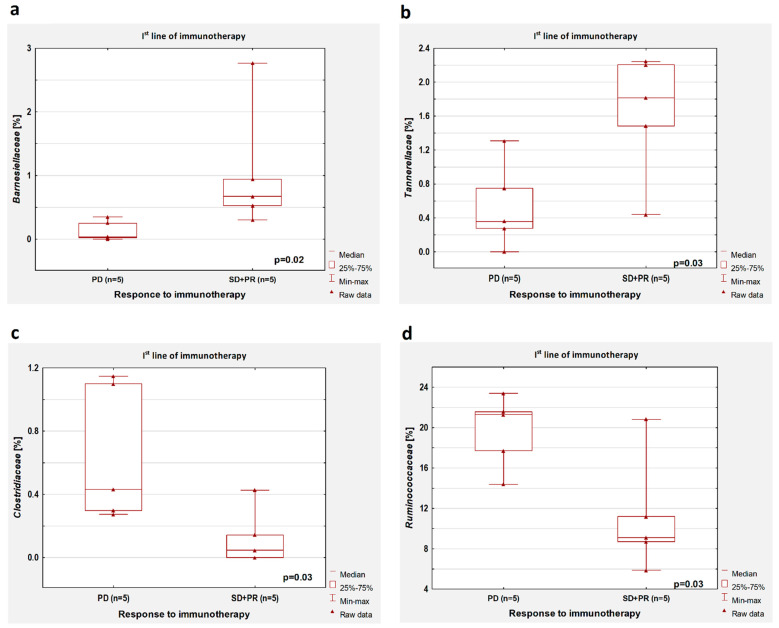
Boxplots showing the content of (**a**) *Barnesiellaceae*, (**b**) *Tannerellaceae*, (**c**) *Clostridiaceae,* and (**d**) *Ruminococcaceae* in the group of patients with the disease control (SD + PR) and progression disease (PD) during first-line immunotherapy. All results concern the patients untreated with antibiotics before immunotherapy.

In patients untreated with antibiotics, a higher percentage of *Barnesiellaceae* (*p* = 0.02, Figure 4a) and *Tannerellaceae* (*p* = 0.03, Figure 4b), as well as a lower abundance of *Clostridiaceae* (*p* = 0.03, Figure 4c) and *Ruminococcaceae* (*p* = 0.03, Figure 4d), were observed in patients with disease control during first-line immunotherapy compared to patients with disease progression.

**Figure 5:**
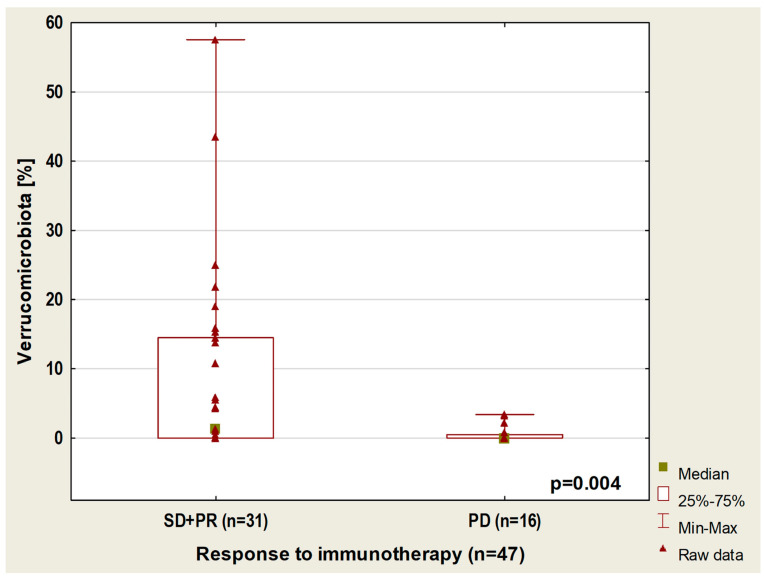
Boxplot showing Verrucomicrobiota content in patients with different responses to immunotherapy.

In the whole group of patients (with or without antibiotic history), we found that the content of *Verucomicrobiota* taxa is higher in patients with disease control compared to those with disease progression regardless of the line of treatment (*p* = 0.004, Figure 5). Moreover, in this group of 47 patients, a higher percentage of *Barnesiellaceae* (*p* = 0.005) and *Tannerellaceae* (*p* = 0.01), as well as a lower abundance of *Clostridiaceae* (*p* = 0.01), were observed in patients with disease control compared to patients with disease progression during first-line immunotherapy. A significantly lower abundance of *Clostridiaceae* was also shown in patients with disease stabilization (SD) or partial response (PR) than in patients with progression disease (PD) regardless of the line of immunotherapy (*p* = 0.02).

### 3.6. Progression-Free Survival

Median PFS in the whole group of patients was 14.3 (95%CI: 8.4 to 28.6) months as described previously [24]. However, in the group untreated with antibiotics, the median PFS was 16.7 months (95% CI: 9.0 to 30.1).

**Figure 6:**
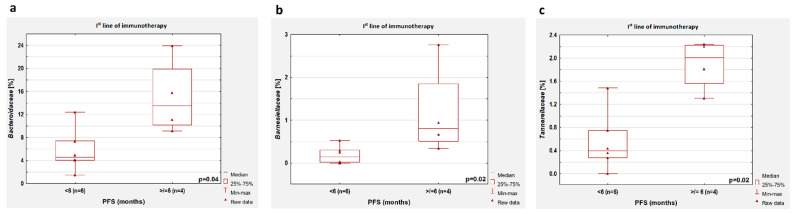
Differences in the abundance of individual bacteria in patients with PFS shorter and longer than 6 months. Boxplots showing the content of (**a**) *Bacteroidaaceae*, (**b**) *Barnesiellaceae,* and (**c**) *Tannerellaceae* in the group of patients treated with first-line immunotherapy.

In the group untreated with antibiotics, we observed a higher percentage of *Bacteroidaaceae*, *Barnesiellaceae,* and *Tannerellaceae* in patients with longer PFS compared to patients with shorter PFS during first-line immunotherapy (*p* = 0.04, *p* = 0.02 and *p* = 0.02, respectively, Figure 6a–c, respectively).

**Figure 7:**
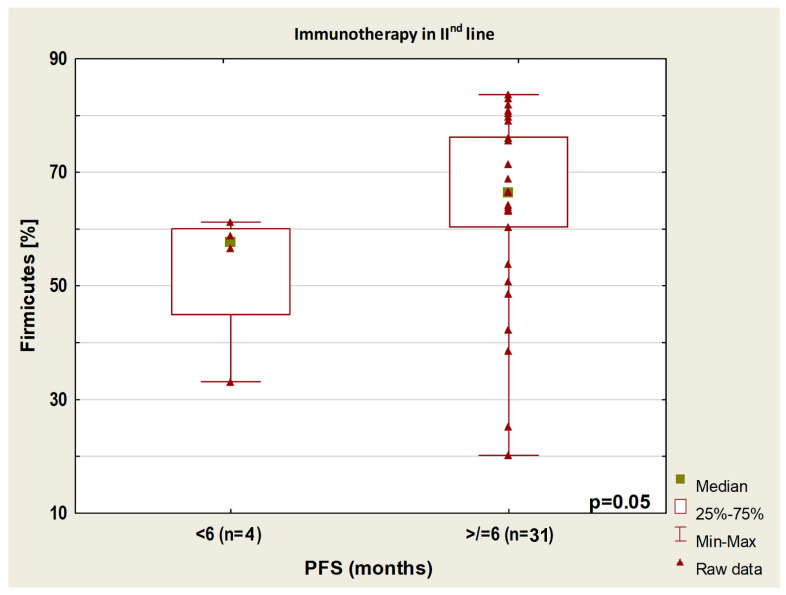
Boxplot showing Firmicutes content in patients with PFS above or below 6 months from the start of immunotherapy.

In patients treated and untreated with antibiotics, the group with longer PFS had a higher abundance of *Barnesiellaceae* than the group with shorter PFS (*p* = 0.01). In patients treated with second-line immunotherapy, the group with PFS higher than 6 months had a higher content of Firmicutes than the group with PFS lower than 6 months (*p* = 0.05, Figure 7).

### 3.7. Overall Survival (OS)

The median OS calculated from the start of immunotherapy was 19.1 months (95%CI: 13.0–25.6). Patients untreated with antibiotics had a median OS of 18.9 months (95%CI: 12.8–26.9). OS did not differ significantly between the groups of patients treated with ICIs in the first- and second-line immunotherapy (*p* > 0.05).

**Figure 8:**
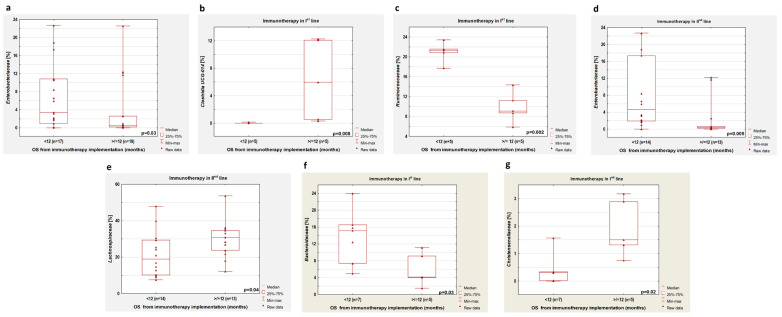
Differences in the abundance of individual bacteria in patients with OS shorter and longer than 12 months. Boxplots showing the content of (**a**) *Enterobacteriaceae* regardless of immunotherapy line; (**b**) *Clostridia UCG-014* in first-line immunotherapy group; (**c**) *Ruminococcaceae* in first-line immunotherapy group; (**d**) *Enterobacteriaceae* in second-line immunotherapy group; (**e**) *Lachnospiracea* in second-line immunotherapy group; (**f**) *Bacteroidaceae* in first-line immunotherapy group; (**g**) *Christensenellaceae* in first-line immunotherapy group. Boxplots (**a**–**e**) show groups untreated with antibiotics before immunotherapy, while boxplots (**f**,**g**) concern patients treated and untreated with antibiotics.

In the group of patients previously untreated with antibiotics, we observed (regardless of the line of immunotherapy) a lower percentage of *Enterobacteriaceae* in patients with OS longer than 12 months compared to patients with OS below one year (*p* = 0.03, Figure 8a). In the first-line immunotherapy group, higher percentages of *Clostridia UCG-014* and a lower content of *Ruminococcaceae* were shown in patients with OS above 12 months compared to patients with OS below this value (*p* = 0.008, Figure 8b and *p* = 0.002, Figure 8c, respectively). In patients who received second-line immunotherapy, an OS greater than 12 months was related to a lower abundance of *Enterobacteriaceae* and a higher percentage of *Lachnospiracea* (*p* = 0.005 and *p* = 0.04, respectively; Figure 8d,e, respectively).

In patients who received first-line immunotherapy and regardless of antibiotic therapy history, we observed a low percentage of *Bacteroidaceae* (*p* = 0.03, Figure 8f) and *Ruminococcaceae* (*p* = 0.002) and a high abundance of *Christensenellaceae* (*p* = 0.02, Figure 8g) and *Clostridia UCG-014* (*p* = 0.002) in persons with longer OS in comparison with patients with shorter OS. The second-line immunotherapy group with longer OS had a higher percentage of *Enterobacteriaceae* compared to the group with shorter OS (*p* = 0.01).

### 3.8. Kaplan-Meier Survival Analysis and Cox Regression Analysis

**Figure 9:**
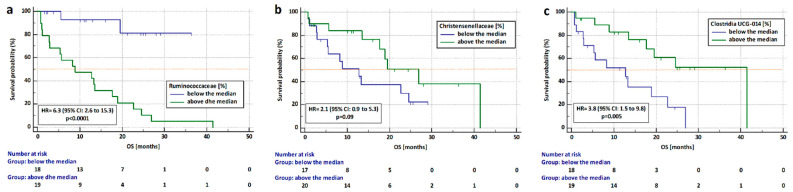
Analysis of overall survival in patients who received Immunotherapy depending on the content of (**a**) *Ruminococcaceae*), (**b**) *Christensenalceae*, and (**c**) *Clostridia UCG-014* regardless of immunotherapy line in the group untreated with antibiotics up to 4 weeks before immunotherapy.

We examined the hazard ratio (HR) for progression and death as well as medians of PFS and OS in patients undergoing immunotherapy depending on the presence of individual bacteria in the gut microbiota (bacteria from our previous analysis and *Clostridia* class). We counted the median percentage of *Barnasiella*, *Tannatellaceae*, *Clostridiaceae*, *Ruminococcaceae*, *Bacterioidaceae*, *Clostridia UCG-014*, *Enterobacteriaceae*, *Lachnospiraceae Anaerovoraceae*, *Bityriciococcaceae*, *Christensenellaceae*, *Peptostreptococcaceae*, *Eubacterium*, and *Tannerellaceae* in a group of patients not previously treated with antibiotics (n = 37). We found that the risk of death is significantly higher in patients with a high abundance of bacteria from the *Ruminococcaceae* family (HR = 6.3, 95% CI: 2.6 to 15.3, *p* < 0.0001) and in patients with a low content of *Clostridia UCG-014* (HR = 3.8, 95% CI: 1.5 to 9.8, *p* = 0.005) regardless of the immunotherapy line (Figure 9a,c, respectively). Patients with a low content of *Christensenellaceae* bacteria had a slightly higher risk of death than patients with a high abundance of this bacteria. (HR = 2.1, 95% CI: 0.87 to 5.3, *p* = 0.09. Figure 9b).

Based on the Cox regression model, we established microbial predictive factors, which affected the risk of death and the risk of progression in patients undergoing immunotherapy. The risk of death was significantly increased by a high content of *Ruminococcaceae* and *Enterobacteriaceae*, and the risk of progression by a high abundance of *Butyriciococcaceae* and *Clostridiaceae,* as well as a low content of *Eubacteriaceae* (Table 1).

## 4. Discussion

In our first analysis of the bacterial phylum, we found that antibiotic treatment up to four weeks before immunotherapy increased the number of Bacteroidota—one of the four main groups residing in the human gut (the other three are Firmicutes, Actinobacteria, and Proteobacteria occupying 65%, 9%, and 5% of the gut microbiome, respectively) [25]. Liang et al. used unsupervised hierarchical clustering at the phylum level with a tumor-agnostic approach (TAA). They observed a lower response rate to immunotherapy in patients enriched in Bacteroidetes and a higher response rate in patients richer in Firmicutes [26]. Furthermore, research showed that systemic antibiotic application was associated with an increase in the Bacteroidetes/Firmicutes ratio and it was linked to poorer immunotherapy outcomes [26]. The tumor-agnostic approach appears to be much needed because studies indicate that one group of bacteria may result in both the presence and lack of response to immunotherapy in different types of cancer. Nevertheless, the examination of the microbiome in patients with particular cancer types separately is still extremely important in the identification of bacterial predictive factors of the effectiveness of anticancer therapies.

Our study showed that patients treated with immunotherapy in the second-line therapy who had PFS longer than six months had a higher content of Firmicutes bacteria. Similarly, Liang et al. indicate that higher Firmicutes content is observed in patients responding to immunotherapy [26]. Moreover, we observed a high abundance of Verucomicrobiota phylum in patients with disease stabilization or partial response to treatment. A member of this group is *Akkermansia mucinifila*. This microorganism is described as a bacterium whose presence in the intestines of cancer patients is closely associated with achieving a response to immunotherapy [18,21,24]. We further found that the application of antibiotics before immunotherapy reduced the content of *Bifidobacteriaceae* and *Clostridia UCG-14* and increased the abundance of *Rikenellaceae*. It has been proven that *Bifidobacteria* are microorganisms that can affect the effectiveness of immunotherapy. Their reduced content may be a negative factor in response to immunotherapy. Longhi et al. highlighted the beneficial role of *Bifidobacteria* in cancer immunotherapy, although the underlying molecular mechanisms remain obscure and still require a great deal of research [21,27,28]. Chau et al. described that the gut microbiome of lung cancer patients who did not develop immune-related adverse events (irAEs) during immunotherapy was relatively enriched in *Bifidobacterium* [29]. In our study, we did not find any significant correlations in treatment response, PFS or OS, and *Bifidobacteria* abundance, but it may be due to the small size of the study group.

Chau et al. proved that responders to combined chemoimmunotherapy showed increased *Clostridiales* abundance. In our study, we noted a reduction of *Clostridia UCG-014* abundance as a result of antibiotic treatment. In addition, we showed that the use of chemotherapy in first-line therapy could also reduce the content of bacteria from this group, which may be unfavorable and cause a higher risk of death in patients treated with second-line immunotherapy. Our observation indicated that *Clostridia UCG-014* was more abundant in patients untreated with antibiotics and with long overall survival on first-line immunotherapy. The size of the study groups was very small (five patients in groups with more and less than 1-year survival), but it appeared that the content of this bacteria may be a strong predictive factor for immunotherapy. However, it also seemed that a higher *Clostridia UCG-014* content was associated with the occurrence of immunotherapy toxicity. *Clostridia UCG-014* should be a subject for further studies. It is a relatively recent group, separated thanks to next-generation sequencing, and its impact on oncology treatment has not been described so far. These bacteria belong to the very broad and heterogeneous *Clostridia* class, among which are pathogenic species as well as bacteria-producing beneficial SCFAs (Short-Chain Fatty Acids). SCFAs-producing bacteria could potentially be used as probiotics. *Butyricoccaceae* are in the SCFAs-producing group. We observed a reduced amount of this group in patients who received first-line chemotherapy, which may affect the effectiveness of ICIs used in the second-line of therapy.

Particular attention should be paid to bacteria of the *Clostridium* genus, as some may improve the efficacy of immunotherapy. However, bacteria in this group may also have unfavorable health effects, including a lack of efficacy in immunotherapy [19,22,30,31]. Tomita et al. indicated that *Clostridium butyricum MIYAIRI 588* (CBM588) improved ICIs efficacy in lung cancer patients [30]. They found that NSCLC patients treated with proton pump inhibitors (PPIs) had significantly reduced efficacy with immune checkpoint inhibitors, but the administration of CBM588 significantly restored the reduced ICIs’ efficacy and improved survival. In addition, *C. butyricum* CBM588 prolonged the overall survival in patients receiving PPIs and antibiotics together [30]. The authors indicated that manipulating commensal microbiota by CBM588 may improve the therapeutic efficacy of ICIs in cancer patients receiving PPIs [30]. Chang et al. conducted the study on butyrate-producing *Butyricicoccus pullicaecorum* (genus *Butyricicoccus*, family *Oscillospiraceae*, order *Eubacteriales,* class *Clostridia*) on mice with 1,2-dimethylhydrazine (DMH)-induced colorectal cancer (CRC). The authors also examined the influence of the microbial metabolite for *B. pullicaecorum* on CRC cells. They showed that the administration of *B. pullicaecorum* or its metabolites improved the clinical outcome of CRC by activating the SCFAs transporter and/or receptor. These results indicate that *B. pullicaecorum* has probiotic and anti-CRC potential [19].

Bacteria of the *Clostridia* class, *Clostridiales* and *Ruminococcaceae*, and their importance in melanoma immunotherapy were examined by Gopalakrishnan et al. [32]. They examined fecal samples from melanoma patients who were undergoing anti-PD-1 immunotherapy and found that the *Clostridiales order* and the *Ruminococcaceae* family were enriched in immunotherapy responders. [32]. In our study, we found a high content of *Clostridiaceae* and *Ruminococcaceae* in non-responding patients with disease progression. Moreover, we observed a higher percentage of *Ruminococcaceae* in patients with overall survival of fewer than 12 months. Furthermore, Kaplan–Meier and Cox analyses confirmed the overabundance of *Ruminoccocaceae* as a risk factor for the reduced time of survival and death. Newsome et al. found in 67 advanced NSCLC patients treated with immunotherapy, that *Butyricicoccus*, *Clostridiales*, and *Lachnoclostridium* (family *Ruminococcaceae*) were the genera associated with a lack of response to immunotherapy [22]. In our Cox regression analysis, we found that a high content of *Butyricciococcaceae and Clostridiacea* and a low content of *Eubacteriaceae* were associated with a high hazard ratio for progression risk. In contrast, a high content of *Ruminococcaceae* and *Enterobacteriaceae* was associated with a high risk of death in patients treated with ICIs regardless of immunotherapy line.

Cheng et al. found that the *Clostridiaceae* family was significantly enriched in cancer patients who did not respond to immunotherapy. Moreover, authors showed that *Archaea*, *Lentisphaerae*, *Victivallaceae*, *Victivallales*, *Lentisphaeria*, *Methanobacteriaceae*, *Methanobacteria*, *Euryarchaeota*, *Methanobrevibacter*, and *Methanobacteriales* were significantly enriched in the response group [33]. It should be noted that Cheng et al. study group was very heterogeneous in terms of cancer type. They enrolled patients treated with immunotherapy and histopathologically confirmed NSCLC, hepatocellular carcinoma, gastric cancer, colorectal carcinoma, melanoma, and others. Moreover, cancer groups were relatively small (from 2 to 14 patients). On the one hand, this may be a disadvantage, since the group was not homogeneous in terms of cancer type. On the other hand, this may be an advantage, since the common point for all cancers was immunotherapy and its effectiveness in different types of cancer. We already mentioned this issue in the publication of Liang et al. with the tumor-agnostic approach in microbiome analysis [26].

Matson et al. evaluated the association between microbiome composition and clinical response to immunotherapy in metastatic melanoma patients and found a higher relative abundance of *Ruminococcus obeum* (genus *Ruminococcus,* family *Oscillospiraceae,* order *Eubacteriales,* class *Clostridia*) and *Roseburia intestinalis* (genus *Roseburia,* family *Lachnospiraceae,* order *Eubacteriales,* class *Clostridia*) in non-responders [27]. Peters et al. investigated the relationship between the gut microbiome and immunotherapy response (anti-PD-1 and anti-CTLA-4) in melanoma patients and found that a high abundance of *Ruminococcus gnavus* was related to shorter PFS [34]. Jin et al., in a study of patients with advanced NSCLC (n = 37) undergoing anti-PD-1 immunotherapy, reported that *unclassified Ruminococcus* was enriched in non-responders [35]. Chaput et al. study indicated that *Ruminococcus* genus abundance was lower when colitis occurs as a complication of ipilimumab treatment in patients with metastatic melanoma [36]. They also indicated that a high content of *Clostridium XIVa* was associated with longer PFS in melanoma patients [36].

We found that two members of the *Clostridia* class, *Lachnospiraceae* and *Christensenellaceae,* were overrepresented in NSCLC patients with overall survival for more than 12 months. McCulloch and colleagues found that *Lachnospiraceae* was closely related to favorable anti-PD-1 clinical response via the enhancement of the immunostimulation against cancer and self-antigens [20]. Furthermore, Cheng et al. found higher *Lachnospiraceae* abundance in advanced cancer patients who responded to anti-PD-1 treatment [33]. Peng et al. indicated that in patients with gastrointestinal cancers (colorectal cancer, esophageal cancer, and gastric cancer) receiving anti-PD-1/PD-L1 treatment, the relative abundance of *Lachnospiraceae* was significantly higher in responders than in non-responders. The above studies and our observations show that the Clostridia class deserves special attention in developing bacterial predictive factors for immunotherapy.

In the literature, we can find information suggesting that the composition of the gut microbiome is related to histopathological diagnosis [24,37,38]. In our study, we observed that a higher abundance of *Peptostreptococcaceae* was observed in previously untreated adenocarcinoma patients compared to SqC patients. Moreover, the content of *Prevotella was* higher in antibiotic-untreated AC compared to SqC patients. Qin et al., in their clinical study (NCT03244605), found that the amount of *Peptostreptococcace* was significantly lower in patients with lung adenocarcinoma compared to healthy subjects. However, they showed no differences in the content of this bacteria between the different stages of adenocarcinoma: Hyperplasia/carcinoma in situ, minimally invasive adenocarcinoma, and invasive carcinoma [39]. They also found that *Prevotella* content was not significantly different between healthy individuals and patients with hyperplasia/carcinoma in situ, while it was higher in minimal invasive adenocarcinoma or invasive adenocarcinoma patients [39]. The authors did not study the differences in microbiome composition between AC and SqC. Differences between lung cancer histologic subtypes may be particularly evident in bacterial composition if the microbiome is examined in tumor tissue or bronchoalveolar lavage [40]. Nevertheless, the ability of bacteria to break the intestinal barrier and migrate to distant organs indicates that it is important to consider which bacteria may indirectly or directly contribute to the development of particular subtypes of lung cancer.

In our study, PFS of more than 6 months was associated with higher content of the *Barnesiellaceae* (order *Bacteroidales*, class *Bacteroidia*) and *Tannerellaceae* (order *Bacteroidales*, class *Bacteroidia*) families in patients previously untreated with antibiotics who received first-line immunotherapy. Daillère et al. indicated that *Barnesiella intestinihominis*, a species belonging to the *Barnesiellaceae* family, accumulated in the colon and promoted the infiltration of IFN-γ-producing γδT lymphocytes in tumors during cyclophosphamide therapy. Moreover, they indicated that patients with advanced lung cancer treated with *Enterococcus hirae* and *Barnesiella intestinihominis* in combination with chemo-immunotherapy showed longer PFS [17]. The presence of the bacteria *Tannerella forsythia*, an anaerobic bacterium of the *Tannerellaceae* family, is implicated in periodontal diseases, and it is associated with the development of esophageal cancer [41]. Moreover, Håheim et al. showed that a low level of antibodies to the oral bacterium *Tannerella forsythia* has been associated with an increased risk of bladder cancer development [42]. Nevertheless, there are no known reports on the effect of the *Tannerella* genus on the efficacy of immunotherapy. Perhaps, as a pathobiont contributing to inflammation, it stimulates the immune system and induces the production of pro-inflammatory cytokines such as IL-1β, IL-6, and TNF-α by T helper cells.

Peng et al. noted that the content of *Bacteroides* (family *Bacteroidaceae*, order *Bacteroidales*, class *Bacteroidia*) was higher in gastrointestinal cancer patients responding to anti-PD-1/PD-L1 immunotherapy compared to patients who did not respond to this treatment [43]. The *Bacteroides* genus belongs to the *Bacteroidaceae* family. In our study, we found that bacteria from this family were more abundant in patients treated with immunotherapy in first-line therapy who had an overall survival of fewer than 12 months compared to patients with OS for more than 12 months. We also noted that *Enterobacteriaceae* were more prevalent in patients with OS less than 12 months treated with second-line immunotherapy. *Ruminococcaceae* and *Enterobacteriaceae* are both associated with the risk of shorter OS and death.

The results regarding microbiome composition and response to immunotherapy can be inconclusive due to differences in the type of cancer, bacterial synergism, patients’ diet, or ethnicity. For this reason, Oh and colleagues asked the question: ‘Can We Use the Gut Microbiome as a Predictive Biomarker for Clinical Response in Cancer Immunotherapy?’ They pointed out in their review paper the inconclusive findings of the baseline microbiome composition (before the implementation of immunotherapy) in various types of cancer [23]. This indicates a strong need for deeper research in this area.

## 5. Conclusions

Our study shows that the composition of the intestinal microbiome is important in the estimation of the immunotherapy effectiveness and the occurrence of ICIs toxicity in patients with advanced non-small cell lung cancer. We also indicated that treatment with antibiotics up to four weeks before the implementation of immunotherapy alters the intestinal microflora. The *Clostridia* class, which appears to be important for the effectiveness of immunotherapy, has a distinct significance in our analyses. We realize that the group of patients in our study was not large, but based on the results, we are inclined to expand the analysis to specific species of the *Clostridia* class. This will be very difficult, as the group is very broad and extremely heterogeneous. Nevertheless, due to the growing interest in this group as probiotic bacteria, it is worth considering whether they are indicators of the efficacy of immunotherapy in cancer patients, which should also be investigated in a tissue-agnostic approach. Moreover, future studies can focus on selected (based on NGS studies) groups of bacteria that are related to treatment efficacy and deepen the study to the species or subspecies level. This will make it possible to determine whether a particular species can be a predictor of immunotherapy efficacy. It may be possible to use methods that are simpler and less expensive than NGS, e.g., qPCR, to determine individual bacteria. This would be more accessible and easier to implement in daily practice in diagnostic and clinical centers.

## Figures and Tables

**Table 1:** Effect of an abundance of selected bacteria in gut microbiome on the risk of death and progression in patients undergoing immunotherapy in Cox multivariate analysis.

Survival	Factor	Coefficient β	*p*-Value	Hazard Ratio (95% CI)
OS	*Ruminococcaceae*	3.0	0.0002	20.8 (4.3 to 100.6)
	*Enterobacteriaceae*	1.1	0.02	3.1 (1.1 to 8.5)
	Overall Model Fit: Chi-squared = 23.2, *p* < 0.0001			
PFS	*Butyriciococcaceae*	2.6	0.001	13.4 (2.7 to 66.1)
	*Clostridiaceae*	1.3	0.03	3.9 (1.1 to 13.2)
	*Eubacteriaceae*	2.0	0.003	7.3 (2.0 to 26.5)
	Overall Model Fit: Chi-squared = 16.8, *p* = 0.0008			

## Data Availability

The datasets generated during and/or analyzed during the current study are available from the corresponding author upon reasonable request.
